# Classifying patients with psoriatic arthritis according to their disease activity status using serum metabolites and machine learning

**DOI:** 10.1007/s11306-023-02079-7

**Published:** 2024-01-24

**Authors:** John Koussiouris, Nikita Looby, Max Kotlyar, Vathany Kulasingam, Igor Jurisica, Vinod Chandran

**Affiliations:** 1 Division of Rheumatology, Psoriatic Arthritis Program, Schroeder Arthritis Institute, University Health Network, Toronto, Canada; 2 Department of Laboratory Medicine and Pathobiology, University of Toronto, Toronto, Canada; 3 Osteoarthritis Research Program, Division of Orthopaedic Surgery, Schroeder Arthritis Institute, University Health Network, Toronto, Canada; 4 Data Science Discovery Centre for Chronic Diseases, Krembil Research Institute, University Health Network, Toronto, Canada; 5 Division of Clinical Biochemistry, Laboratory Medicine Program, University Health Network, Toronto, Canada; 6 Department of Computer Science, University of Toronto, Toronto, Canada; 7 Department of Medical Biophysics, University of Toronto, Toronto, Canada; 8 Division of Rheumatology, Department of Medicine, University of Toronto, Toronto, Canada; 9 Institute of Medical Science, University of Toronto, Toronto, Canada; 10 Krembil Research Institute, University Health Network, Toronto, Canada

**Keywords:** Lipids, Psoriasis, Psoriatic arthritis, Metabolites, Machine learning

## Abstract

**Introduction:**

Psoriatic arthritis (PsA) is a heterogeneous inflammatory arthritis, affecting approximately a quarter of patients with psoriasis. Accurate assessment of disease activity is difficult. There are currently no clinically validated biomarkers to stratify PsA patients based on their disease activity, which is important for improving clinical management.

**Objectives:**

To identify metabolites capable of classifying patients with PsA according to their disease activity.

**Methods:**

An in-house solid-phase microextraction (SPME)—liquid chromatography-high resolution mass spectrometry (LC-HRMS) method for lipid analysis was used to analyze serum samples obtained from patients classified as having low (n = 134), moderate (n = 134) or high (n = 104) disease activity, based on psoriatic arthritis disease activity scores (PASDAS). Metabolite data were analyzed using eight machine learning methods to predict disease activity levels. Top performing methods were selected based on area under the curve (AUC) and significance.

**Results:**

The best model for predicting high disease activity from low disease activity achieved AUC 0.818. The best model for predicting high disease activity from moderate disease activity achieved AUC 0.74. The best model for classifying low disease activity from moderate and high disease activity achieved AUC 0.765. Compounds confirmed by MS/MS validation included metabolites from diverse compound classes such as sphingolipids, phosphatidylcholines and carboxylic acids.

**Conclusion:**

Several lipids and other metabolites when combined in classifying models predict high disease activity from both low and moderate disease activity. Lipids of key interest included lysophosphatidylcholine and sphingomyelin. Quantitative MS assays based on selected reaction monitoring, are required to quantify the candidate biomarkers identified.

**Supplementary Information:**

The online version contains supplementary material available at 10.1007/s11306-023-02079-7.

## Introduction

Psoriasis is an inflammatory skin disease affecting approximately two percent of the global population (Lebwohl et al., 2014). Approximately 25% of individuals with psoriasis present with an inflammatory arthritis known as psoriatic arthritis (PsA) (Alinaghi et al., 2019). This condition is linked to diminished quality of life and impaired functional capacity (van der Heijde et al., 2020). PsA is characterized by various clinical manifestations such as peripheral joint inflammation, enthesitis, tendonitis, dactylitis, inflammatory spinal disease and non-musculoskeletal manifestations (FitzGerald & Gladman, 2018).

The key to improving outcome in PsA is effective therapy based on a valid assessment of disease activity for each patient (Haroon et al., 2015); however, this is difficult because the presence and severity of various clinical features vary among patients and within each patient over time (Perruccio et al., 2020). Acute phase reactants such as C-reactive protein, which are traditionally elevated during inflammatory states are often within normal range in many patients despite active disease and are thus unreliable markers of PsA disease activity (Perruccio et al., 2020). Composite disease activity measures such as the psoriatic arthritis disease activity score (PASDAS)—a validated clinical method of assessing PsA activity- have been developed; however, these scores are influenced by assessor variability and confounding comorbidities (Perruccio et al., 2020). Valid and reliable biomarkers that can stratify PsA patients based on their disease activity are important for improving clinical management.

Metabolomics provides a powerful platform for identifying biomarkers for a complex, multifactorial disease such as PsA. A scoping literature review revealed that very few studies have explored the association between the metabolome and PsA activity and of those that have, they used only small sample sizes of less than fifty participants (Koussiouris et al., 2021). Using serum samples, these studies found trimethylamine-N-oxide, eicosanoids and fatty acids to be associated with PsA activity (Koussiouris et al., 2021; Looby et al., 2021). One of these preliminary studies was an untargeted metabolomics analysis of serum samples obtained from individuals with varying PsA activity (mild, moderate, severe; n = 10 each) and healthy controls (n = 10) conducted at our facility (Looby et al., 2021). Patients with severe PsA had elevated levels of select long-chain fatty acids and the fatty acid 1,11-undecanedicarboxylic acid was identified as a classifier of PsA patients vs. healthy individuals (Looby et al., 2021). There is a need to validate these findings as well as identify additional lipids that can classify PsA disease activity using larger patient cohorts.

In this study, we sought to identify metabolites capable of classifying patients with PsA according to their disease activity as measured by PASDAS, using an in-house solid-phase microextraction (SPME)—liquid chromatography–mass spectrometry (LC–MS) method for lipid analysis on 372 PsA serum samples. To the best of our knowledge, this is the first time this type of research has been completed on this magnitude of patient samples.

## Materials and methods

### Materials

The following materials were used for thin film device preparation, sample preparation and instrumental analysis. Thin film stainless steel combs and the Concept-96 were purchased from PAS technologies (Magdala, Germany). The dip coater and lab oven were purchased from Ni-Lo Scientific (Ottawa, Canada) and Hogentogler (Columbia, USA), respectively. Oasis hydrophilic-lipophilic balanced (HLB) particles (50–65 μm) and polystyrene divinylbenzene with weak anion exchanger (PS-DVB-WAX) particles (50–65 μm) were purchased from Waters Corporation (Milford, USA). HPLC and LC–MS grade solvents (acetonitrile, methanol, water, isopropanol and acetone), concentrated hydrochloric acid, N,N-dimethylformamide (DMF), L-ascorbic acid and dodecanedioic acid were purchased from Thermo Fisher Scientific (New Waltham, USA). The following internal standards and chemicals were purchased from Millipore Sigma (Burlington, USA): amphetamine-d5, MDMA-d5, ketamine-d4, diazepam-d5, oxazepam-d5, codeine-d3, fentanyl-d5, heroin-d9, buprenorphine-d4, nordiazepam-d5, SPLASH LIPIDOMIX Mass Spec Standard and polyacrylonitrile. 1 mL deep-well plates were purchased from Canadian Life Science (Peterborough, Canada). The standards 1,11-undecanedicarboxylic acid and 10-hydroxy-2-decenoic acid were purchased from Toronto Research Chemicals (Toronto, Canada). Adrenic acid, arachidonic acid, di-hommo-gamma-linolenic acid, docosahexaenoic acid, docosapentaenoic acid, eicosapentaenoic acid, linolenic acid, gamma-linolenic acid, stearidonic acid, alpha-linolenic acid, arachidic acid, lignoceric acid, nervonic acid and palmitoleic acid were purchased from Cayman Chemical (Ann Arbor, USA). 

### Patients

Serum samples were obtained from the University of Toronto Psoriatic Disease Research Program Biobank. These samples were from PsA patients not previously treated with advanced targeted therapies and with no active infection or history of cancer. Sample collection took place from 2008 to 2020. The study was approved by the University Health Network Research Ethics Board (#21-5311). Patients were evaluated using a standard protocol that included clinical assessment by a rheumatologist, patient reported outcomes and routine laboratory methods. Based on these assessments, the psoriatic arthritis disease activity score (PASDAS), ranging from 0 to 10, with 10 indicating most active disease was calculated, and patients were classified based on validated cut-off scores as low (< 3.2), moderate (3.2–5.4) and high disease (> 5.4) activity (Helliwell et al., 2013; Perruccio et al., 2020). Table 1 summarizes patient demographics. The following number of samples were obtained from our biobank from PsA patients with low (n = 134), moderate (n = 134), and high (n = 104) disease activity. The sample size was calculated to provide 80% power to draw the conclusion that the multivariate model to distinguish low versus moderate-to-high disease activity has an AUC larger than 0.85 when the true AUC is 0.9 is at a significance level of 0.05.

**Table 1:** Patient demographics

Characteristic	Low	Moderate	High
PASDAS range	< 3.2	3.2–5.4	> 5.4
Number of patients	134	134	104
Percentage of females	44	43	50
Mean age in years (SD)	53.9 (12.3)	53.2 (12.8)	50.9 (13.4)
Mean PsA duration in years (SD)	13.9 (11.6)	13.8 (12.3)	12.4 (11.7)
Mean PASI (SD)	2.1 (2.6)	4.2 (6.5)	5.6 (8.3)
Mean CRP (SD)	7.3 (11.5)	6.8 (8.1)	12.8 (19.1)
Mean swollen joint count (SD)	0.2 (0.6)	0.8 (2.0)	5.1 (6.3)
Mean active* joint count (SD)	0.5 (1.3)	2.2 (3.9)	11.0 (9.5)
Percentage of patients with enthesitis	2.9	10.4	40.4
Percentage of patients with dactylitis	0	2.9	27.8
Percentage of patients treated with NSAIDs	57	62	58
Percentage of patients treated with steroids	0	4	10
Percentage of patients treated with DMARDs	54	58	64
Percentage of patients with comorbidities	Diabetes (7), Hyperlipidemia (30), High cholesterol (30), High Triglycerides (7), Vascular disease (0)	Diabetes (16), hyperlipidemia (34), high cholesterol (35), high triglycerides (14), vascular disease (0)	Diabetes (15), hyperlipidemia (22), high cholesterol (21), high triglycerides (10), vascular disease (4)

*PASDAS* psoriatic arthritis disease activity score, *SD* standard deviation, *PsA* psoriatic arthritis, *PASI* psoriasis area and severity index, *CRP* C-reactive protein, *NSAIDs* non-steroidal anti-inflammatory drugs, *DMARDs* disease-modifying antirheumatic drugs

*Swollen or tender

### Thin film microextraction device preparation

The thin film microextraction (TFME) device was prepared using a dip-coating method optimized in the Schroeder Arthritis Institute–Centre for Arthritis Diagnostic and Therapeutic Innovation: Metabolomics Core Facility. First, the stainless-steel blades were prepared using a previously established protocol (Mirnaghi & Pawliszyn, 2012). Stainless steel blades were etched for 1 h in concentrated hydrochloric acid and then thoroughly rinsed in tap water. After rinsing, the blades were dried in an oven overnight. A 7% w/v polyacrylonitrile (PAN) solution was then prepared in N,N-dimethylformamide (DMF). A specialized software-operated dip-coating machine was used to coat the stainless-steel support with a slurry mixture consisting of 7% w/v 1:1 HLB and PS-DVB-WAX particles in PAN solution. After the application of each layer, the coating was placed in an oven to cure at 150 °C for 1 min. The solution was used to coat a total of 32 combs, each of which had 12 blades. A TFME brush was assembled using every 8 combs once the coatings had been applied. The brush was compatible with 96-well plates and used for high throughput sample preparation. The final coating dimensions on each blade was on average 2 mm thick and 2 cm long. Each brush was then cleaned with 50:25:12.5:12.5 (v/v) water:methanol:acetonitrile:isopropanol.

### Sample preparation

Blood was collected in red top serum separator tubes without additives, allowed to clot at room temperature before centrifugation at 2000 g for 15 min. Serum was aliquoted in 0.5 ml vials and frozen at − 80 °C until analysis. Serum samples were processed using TFME, which consisted of a thin stainless-steel blade that had been coated with a mixture of HLB and PS-DVB-WAX particles. Each well of a 96-well plate was filled with 200 μL of serum and 400 μL of phosphate buffered saline (PBS) containing deuterated nordiazepam. The serum samples were then agitated for 30 min at 500 rpm prior to extraction with SPME. The following SPME protocol was performed under ambient conditions. During the sample equilibration time, the TFME device was placed onto the Concept-96 manual kit and was conditioned in a mixture of 1:1 methanol:water (v/v) for 30 min at 1500 rpm. Following conditioning, the device was rinsed in water for 15 min at 1500 rpm and then exposed to the serum samples for a period of 1 h at 1500 rpm. After the extraction process, the device was rinsed in 90:5:5 water:methanol:acetone (v/v/v) for 10 s at 500 rpm to remove any loosely attached matrix components from its surface. Finally, the extracted metabolites were desorbed in 600 μL of 4:3:3 methanol:acetonitrile:water (v/v/v) + 0.1% ascorbic acid containing deuterated amphetamine, MDMA, ketamine, diazepam, oxazepam, codeine, fentanyl, heroin, buprenorphine and SPLASH LIPIDOMIX Mass Spec Standard, for 1 h at 1500 rpm. 200 μL of the desorption solution was then diluted with 400 μL of 9:6:5 isopropanol/acetonitrile/methanol to produce a final extract composed of 3:3:3:1 isopropanol:methanol:acetonitrile:water, thus ensuring compatibility with the chromatographic conditions of the LC–MS metabolomics method that would be used for analysis. A pooled quality control (QC) sample was prepared by combining 10 μL of each sample extract in a separate well. The pooled QC was injected approximately after every 10 sample injections.

### Instrumental analysis: liquid chromatography coupled with high-resolution mass spectrometry

High performance liquid chromatography (HPLC) with high-resolution mass spectrometry (HRMS) detection was performed using a Vanquish autosampler and Vanquish binary pump coupled to a Q Exactive Plus Hybrid Quadrupole-Orbitrap Mass Spectrometer (Thermo Fisher Scientific, Waltham, USA). Chromatographic separation was conducted on an Accucore C30 HPLC column (100 mm × 2.1 mm, 2.6 μm), purchased from Thermo Fisher Scientific, (Waltham, USA). Gradient elution was achieved over 30 min in positive mode using mobile phases consisting of 99.9/0.1 water/formic acid (v/v) and 99.9/0.1 methanol/formic acid (v/v). For negative-mode chromatography, gradient elution was achieved over 20 min using mobile phases consisting of 99.9/0.1 water/formic acid (v/v) and methanol. The flow rate was 0.4 mL/min in both positive and negative mode. Sample extracts were injected at a volume of 5 μL and the autosampler and column temperature were maintained at 5 °C and 60 °C, respectively. Further details on the gradient elution used for chromatography can be found in Online Resource 1.

The Q Exactive Plus Hybrid Quadrupole-Orbitrap Mass Spectrometer was equipped with an Ion Max heating source, which contained a heated electrospray ionization (HESI-II) probe. Mass spectrometer parameters were optimized based on direct infusion with SPLASH LIPIDOMIX Mass Spec Standard. The mass spectrometer was run in positive mode at high resolution (70,000) and data was acquired within an m/z range of 150–1200 with an automatic gain control target of 1E6 and an injection time of 100 ms. In negative mode, the mass spectrometer was run at high resolution (70,000), and data was acquired within an m/z range of 75–1000 with an automatic gain control target of 1E6 and an injection time of 50 ms. In both modes, a full MS scan and data-dependent-MS2 scan (Top 5) with iterative exclusion was run on the pooled QC’s, pooled PsA-low, pooled PsA-moderate and pooled PsA-high. The sheath, auxiliary and sweep gas were set to 48, 11 and 1, respectively in positive mode and 75, 30 and 1, respectively in negative mode. The electrospray voltage applied for positive mode was 3.50 kV and − 2.60 kV in negative mode.

SPLASH LIPIDOMIX Mass Spec Standard containing a mixture of deuterium labeled lipids (15:0–18:1(d7) PC, 18:1(d7) Lyso PC, 15:0–18:1(d7) PE, 18:1(d7) Lyso PE, 15:0–18:1(d7) PG, 15:0–18:1(d7) PI, 15:0–18:1(d7) PS, 15:0–18:1(d7)-15:0 TAG, 15:0–18:1(d7) DAG, 18:1(d7) MAG, 18:1(d7) Chol Ester, d18:1–18:1(d9) SM, 15:0–18:1(d7) PA, Cholesterol-d7) and several fatty acid standards (dodecanedioic acid, 11-undecanedicarboxylic acid, 10-hydroxy-2-decenoic acid, adrenic acid, arachidonic acid, di-hommo-gamma-linolenic acid, docosahexaenoic acid, docosapentaenoic acid, eicosapentaenoic acid, linolenic acid, gamma-linolenic acid, stearidonic acid, alpha-linolenic acid, arachidic acid, lignoceric acid, nervonic acid, palmitoleic acid) were used as quality control standards throughout the LC-HRMS analysis. The standards were prepared in 3:3:3:1 isopropanol/methanol/acetonitrile/water at 1% v/v and 200 ppb, respectively.

### Data pre-processing and statistical analysis

LC-HRMS data files acquired during instrumental analysis were pre-processed and analyzed using Compound Discoverer 3.3 (Thermo Fisher Scientific, New Waltham, USA). The raw LC-HRMS data files were loaded onto Compound Discoverer and automatically processed using the default parameters of the workflow: “Untargeted Metabolomics with Statistics, Detect Unknowns with ID using Online Databases and mzLogic.” The following steps were included in this workflow. Spectra were selected from raw data followed by retention time (RT) alignment with RT tolerance of 0.2 min and 5 ppm mass precision. Features with a sample/blank peak-intensity ratio of < 3 were labeled as background compounds. Pooled quality control (QC) samples were prepared by combining 10 μL of each sample extract and injected approximately every 10 sample injections. They were used to correct for time-dependent batch effects using SERRF (systematic error removal with random forests) (Fan et al., 2019).

Processed metabolite data were analyzed using eight machine learning methods to predict disease activity levels: adaptive boosting (AdaBoost), decision tree (J48), LogitBoost, logistic regression, logistic regression with L1 (Lasso) regularization, logistic regression with L2 (Ridge) regularization, Naïve Bayes, random forest and support vector machine (SVM), as implemented in the mlr 2.15.0 package (Bischl B). Predictions were evaluated using ten-fold cross-validation. Receiver operating characteristic (ROC) from predictions were plotted using the pROC 1.15.3 package (Robin et al., 2011). P-values of predictions were calculated using the verification 1.42 package (N-RA, 2015). Each model included several features, ranging from two to eighty and a combination of features achieved better classification. Top performing methods were selected based on area under the curve (AUC) and significance. An AUC of 0.7 was chosen as a lowest cut-off for clinical usability; however, a clinically usable diagnostic test would require an AUC of > 0.9 (Chandran, 2020).

For each classification of PsA disease activity, significant features across all methods with AUC > 0.7 were annotated. On Compound Discoverer, Chemspider was used to annotate features based on exact mass and predicted formula. We used all available source databases, including BioCyc (Caspi et al., 2016), Human Metabolome Database (Wishart et al., 2007), KEGG database (Kanehisa et al., 2017) and Serum Metabolome Database (Psychogios et al., 2011). mzCloud on Compound Discoverer, as well as global natural products social molecular networking (GNPS) version 5.1 (Wang et al., 2016) and MS-DIAL online (Tsugawa et al., 2015) were used to annotate compounds from MS/MS spectra. All features that overlapped across significant models for each comparison were reported.

## Results

### Low versus moderate disease activity

**Fig. 1:**
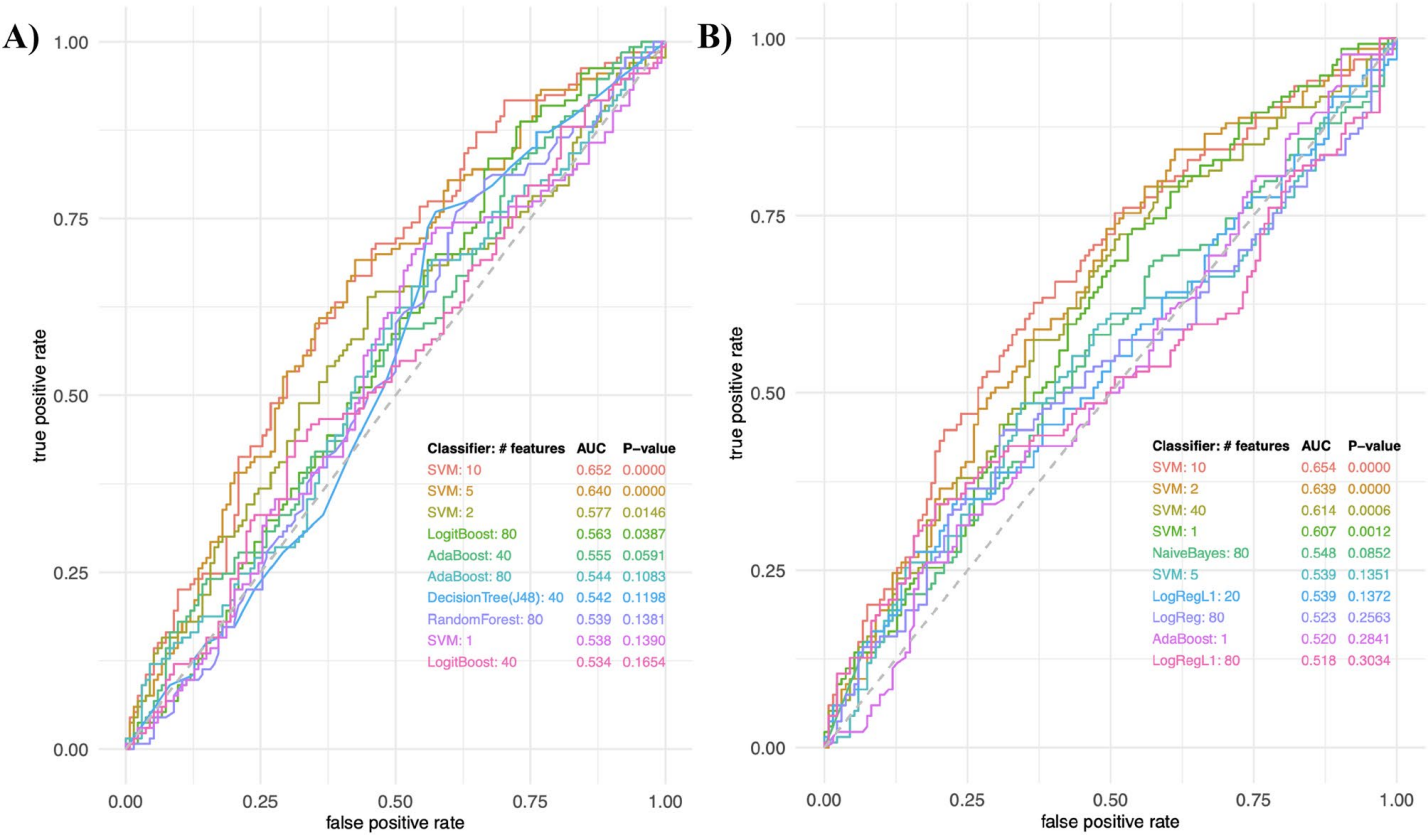
Top performing predictive models for low versus moderate disease activity. **A** ROC curves of predictive feature combinations using various classifiers in positive mode. **B** ROC curves of predictive feature combinations using various classifiers in negative mode. SVM support vector machine, AdaBoost adaptive boosting, LogReg logistic regression, LogRegL1 logistic regression with L1 (Lasso) regularization

Principal component analysis was employed to visualize the dataset (see Online Resource 2). The pooled quality control samples clustered, indicating instrumental stability during acquisition. Machine learning classification algorithms were used to predict moderate disease activity patients from low disease activity patients using a combination of features. As shown in Fig. 1, no model achieved an AUC greater > 0.7; thus, we did not pursue identification of these features.

### Low versus high disease activity

**Fig. 2:**
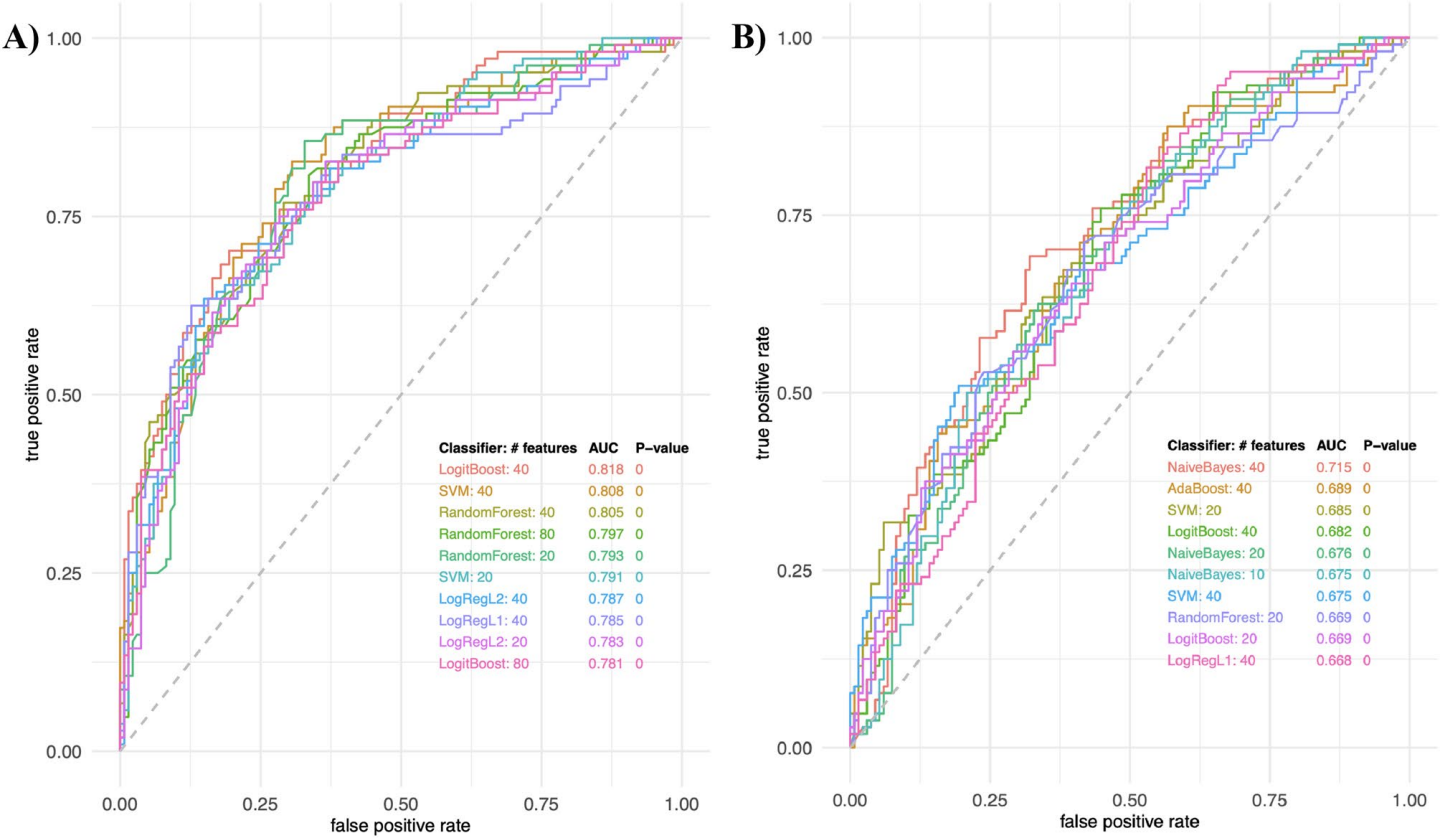
Top performing predictive models for low vs. high disease activity. **A** ROC curves of predictive feature combinations using various classifiers in positive mode. **B** ROC curves of predictive feature combinations using various classifiers in negative mode. SVM support vector machine, LogRegL2 logistic regression with L2 (Ridge) regularization, LogRegL1 logistic regression with L1 (Lasso) regularization, AdaBoost adaptive boosting

Machine learning classification algorithms were used to predict high disease activity patients from low disease activity patients using combination of features. As shown in Fig. 2, the best model achieved AUC 0.818. We successfully tentatively identified 45% features included in our models, and of those, we were able to confirm 29% of compounds using MS/MS spectra. See Table 2 for a complete list of confirmed metabolites in positive and negative mode. See Online Resource 1 for tentatively identified compounds. See Online Resource 2 for extracted ion chromatograms and MS/MS spectra for confirmed compounds.

**Table 2:** Confirmed compounds in the top performing predictive models for low vs. high disease activity

m/z	RT (min)	Confirmed compound	Adduct	Biochemical importance
202.0473	2.96	Hippuric acid	[M + Na]^+^	Uremic toxin
205.0971	2.32	DL-tryptophan	[M + H]^+^	Indolyl carboxylic acid
542.3240	11.66	LysoPC(20:5(5Z,8Z,11Z, 14Z,17Z))	[M + H]^+^	Lysophosphatidylcholine. Pro-inflammatory properties and pathological component in atherosclerotic lesions
703.5744	19.32	Sphingomyelin (d18:1/16:0)	[M + H]^+^	Sphingolipid found in animal cell membranes
178.0513	3.98	4-acetamidobenzoic acid	[M-H]^−^	Benzoic acid. Considered part of the human exposome
263.1042	4.08	N-phenylacetylglutamine	[M-H]^−^	Accumulates in uremia

### Moderate versus high disease activity

**Fig. 3:**
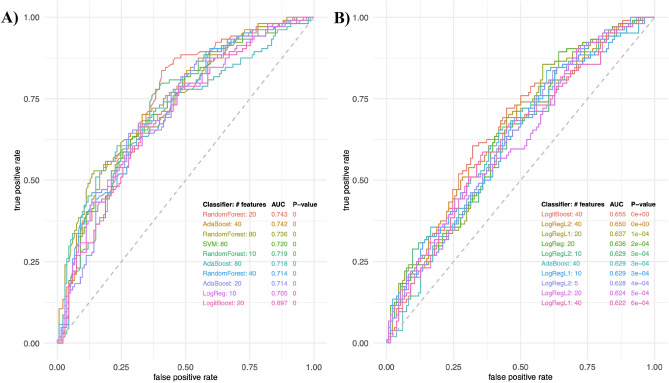
Top performing predictive models for moderate vs high disease activity. **A** ROC curves of predictive feature combinations using various classifiers in positive mode. **B** ROC curves of predictive feature combinations using various classifiers in negative mode. AdaBoost adaptive boosting, SVM support vector machine, LogReg logistic regression, LogRegL2 logistic regression with L2 (Ridge) regularization, LogRegL1 logistic regression with L1 (Lasso) regularization

Machine learning classification algorithms were used to predict high disease activity patients from moderate disease activity patients. The top performing predictive models using combinations of predictive features are shown in Fig. 3; the best model achieved AUC 0.743 in the positive mode. We successfully tentatively identified 45% features included in our models, and of those, we were able to confirm 19% of compounds using MS/MS spectra. See Table 3 for a complete list of confirmed compounds in positive and negative mode. See Online Resource 1 for tentatively identified compounds. See Online Resource 2 for extracted ion chromatograms and MS/MS spectra for confirmed compounds.

**Table 3:** Confirmed compound in the top performing predictive models for moderate versus high disease activity

m/z	RT (min)	Confirmed compound	Adduct	Biochemical importance
256.1095	3.58	Hydroxy bupropion	[M + H]^+^	Alkyl-phenylketone. Detected in several different foods
542.3240	11.66	LysoPC(20:5(5Z,8Z,11Z,14Z,17Z))	[M + H]^+^	Lysophosphatidylcholine. Pro-inflammatory properties and pathological component in atherosclerotic lesions
568.3397	12.53	LysoPC(22:6(4Z,7Z,10Z,13Z,16Z,19Z)/0:0)	[M + H]^+^	Lysophosphatidylcholine. Pro-inflammatory properties and pathological component in atherosclerotic lesions
784.5843	20.13	Arachidonoyl thio-PC	[M + H]^+^	Organonitrogen compound
178.0513	3.98	4-acetamidobenzoic acid	[M-H]^−^	Benzoic acid. Considered part of the human exposome
298.9436	6.15	Perfluorobutanesulfonic acid	[M-H]^−^	Perfluoroalkyl sulfonic acid

### Low versus moderate and high disease activity

**Fig. 4:**
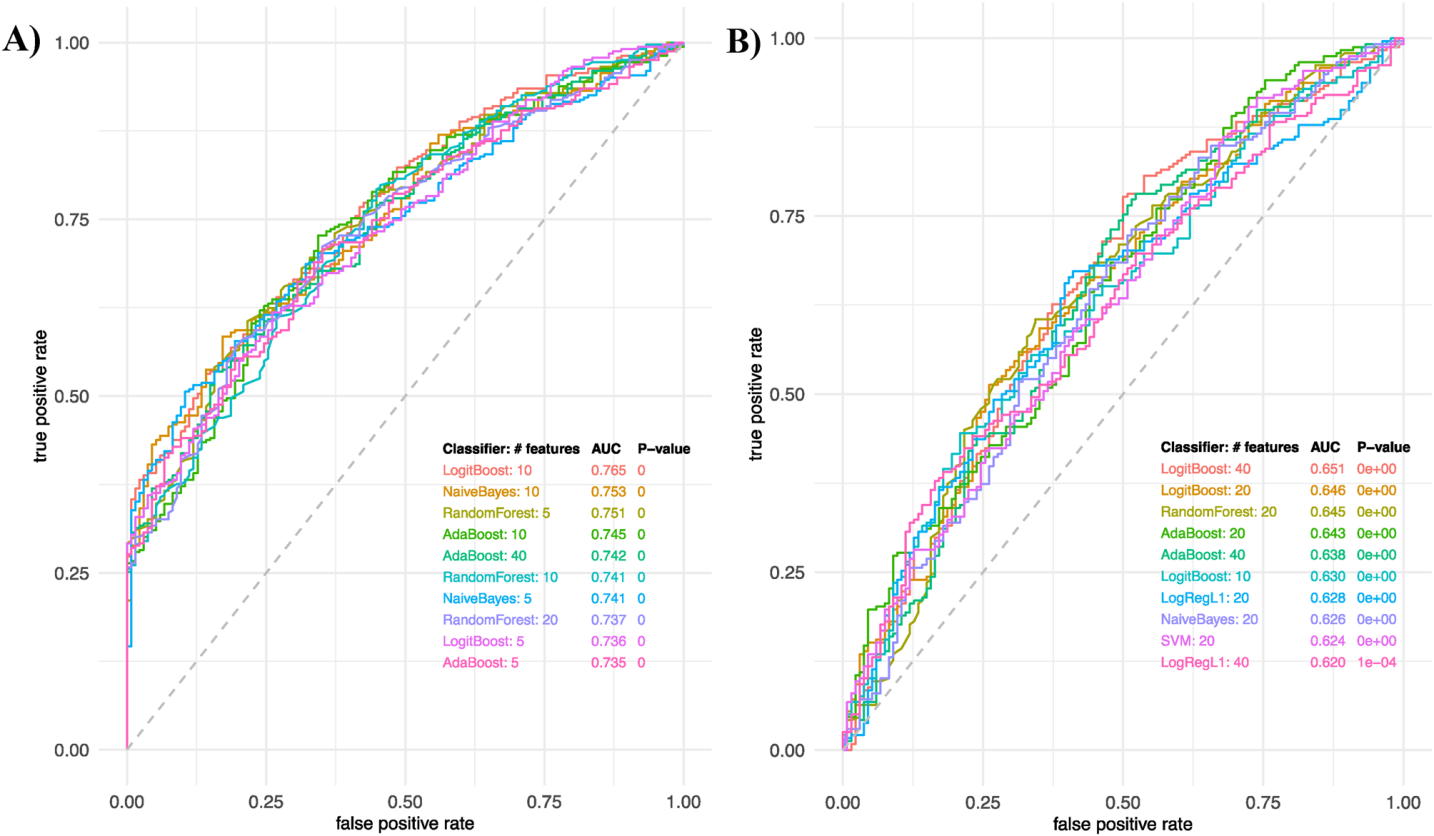
Top performing predictive models for low versus moderate and high disease activity. **A** ROC curves of predictive feature combinations using various classifiers in positive mode. **B** ROC curves of predictive feature combinations using various classifiers in negative mode. AdaBoost adaptive boosting, SVM support vector machine, LogReg logistic regression, LogRegL2 logistic regression with L2 (Ridge) regularization, LogRegL1 logistic regression with L1 (Lasso) regularization

Since having moderate-high disease activity indicates need for escalating treatment in patients with PsA, we also used machine learning classification algorithms to predict moderate and high disease activity patients from low disease activity patients. The top performing predictive models using combinations of predictive features are shown in Fig. 4; the best model achieved AUC 0.765 in the positive mode. We successfully tentatively identified 45% features included in our models, and of those, we were able to confirm 27% of compounds using MS/MS spectra. See Table 4 for a complete list of confirmed compounds in positive mode. See Online Resource 1 for tentatively identified compounds. See Online Resource 2 for extracted ion chromatograms and MS/MS spectra for confirmed compounds.

**Table 4:** Confirmed compound in the top performing predictive models for low vs. moderate and high disease activity

m/z	RT (min)	Confirmed compound	Adduct	Biochemical importance
166.0863	1.40	L-phenylalanine	[M + H]^+^	Alpha-amino acid
188.0706	2.33	Trans-3-indoleacrylic acid	[M + H]^+^	Indole compound
205.0971	2.32	DL-tryptophan	[M + H]^+^	Indolyl carboxylic acid
703.5744	19.32	Sphingomyelin (d18:1/16:0)	[M + H]^+^	Sphingolipid found in animal cell membranes
784.5843	20.13	Arachidonoyl thio-PC	[M + H]^+^	Organonitrogen compound

## Discussion

In this study, we applied an SPME-LC-HRMS untargeted metabolomics method developed in-house to serum samples collected from PsA patients with low, moderate and high disease activity. In a multifactorial, heterogeneous disease such as PsA, a combination of multiple “weak” individual markers into a single ‘‘strong’’ multivariate model may provide better prediction. Thus, we aimed to identify a combination of metabolites that can predict global PsA disease activity, with an area under the curve (AUC) of 0.7 or greater—our arbitrary (minimal usability) cut-off. We identified several lipids, including lysophosphatidylcholine and sphingomyelin, and other metabolites that when combined in classifying models predict high disease activity from both low and moderate disease activity.

We first attempted to differentiate moderate disease activity from low disease activity in both positive and negative mode. No model generated an AUC > 0.7; hence the features were not explored further. Next, we sought to examine the ability of the models to differentiate high disease activity from low disease activity, which would be expected to be easier, considering the greater clinical differences between the two patient groups. The models in both positive and negative mode required twenty or forty features to surpass the 0.7 AUC threshold. We tentatively identified twenty-one features across our positive mode and negative mode models, and of those we confirmed six using MS/MS spectra validation (see Online Resource 2 for MS/MS spectra). Selected features included metabolites from diverse compound classes such as fatty acids, peptides, phosphatidylcholines, and phosphatidylethanolamines. There were also features identified as being part of the human exposome, such as metabolites detected in food. Drugs used for treatment were also detected, as patients were not naïve to treatment. We also considered classifying moderate disease activity patients from high disease activity patients. Our models in positive mode reached a threshold AUC = 0.7 with as little as ten features, which was fewer than in the previous models classifying low vs high disease activity. Tentatively identified features included several phosphatidylcholines and sphingolipids. We were able to confirm four features, of which, two were lysophosphatidylcholines, which were above-mentioned in our low vs high models. Lastly, we classified low disease activity patients from moderate and high disease activity patients since a state of moderate or high disease activity would indicate need to escalate therapy. Our models in positive mode reached a threshold AUC of 0.7 with as little as five features, which was fewer than in the previous models. Multiple phosphatidylcholines and acylcarnitines were tentatively identified. Three out of the five confirmed compounds (DL-Tryptophan, Sphingomyelin (d18:1/16:0) and Arachidonoyl Thio-PC) were also identified in our previous models. Although all identified features may be important in helping to classify PsA disease activity, we focused our attention on compounds that had their identity confirmed using MS/MS.

One of the features confirmed was hippuric acid, a derivative of benzene that is a normal component of urine. Hippuric acid is accumulated in uremic plasma and is thus classified as a uremic toxin (Duranton et al., 2012). There have been a few metabolomics studies that have explored the role of hippuric acid in rats with adjuvant-induced arthritis. Hippuric acid was identified as a potential biomarker in the urine of these rats and researchers found that the compound inhibited bone resorption in vitro, suggesting a possible relationship between this compound and rheumatoid arthritis (RA) (Jiang et al., 2016; Zhao et al., 2020). In humans, Kapoor et al. (2013) identified increased urinary levels of hippuric acid in patients with RA and PsA after treatment with infliximab, an anti-tumor necrosis factor (anti-TNF) agent (Kapoor et al., 2013). In our current study, samples were selected from PsA patients not previously treated with anti-TNF agents. Our data suggest that hippuric acid may be an important urinary and blood metabolite involved in autoimmune/inflammatory arthritis beyond its relationship with treatment.

Additionally, tryptophan, an essential alpha-amino acid was a confirmed compound included in our positive mode models, comparing low and high disease activity. Several metabolomics studies over the last century have examined the role of tryptophan in inflammatory diseases (Yang et al., 2021). Changes in the serum concentration of tryptophan have been reported in patients with PsA and the related disease ankylosing spondylitis, after starting and stopping treatment with anti-inflammatory agents (Aylward & Maddock, 1974). Tryptophan has also been reported at higher concentrations in the serum and synovial fluid of PsA patients compared to RA patients (Bertazzo et al., 1999). More recent studies have pointed to a potential role of tryptophan and its metabolites in regulating inflammation (Sorgdrager et al., 2019). There is a discrepancy among recent metabolomics studies regarding levels of tryptophan in psoriatic disease (Koussiouris et al., 2021). One study found elevated levels of tryptophan in the plasma of psoriasis patients compared to healthy controls, whereas another reported the opposite in serum (Castaldo et al., 2020; Li et al., 2019).

Lipids have been reported to be associated with PsA disease activity, in multiple metabolomic studies (Koussiouris et al., 2021; Looby et al., 2021). For example, a recent study by Coras et al. identified several arachidonic acid-derived oxylipins elevated in PsA patients with enthesitis compared to those without (Coras et al., 2021). Our models included lysophosphatidylcholine (LPC), an inflammatory lipid, thought to be involved in several immune-mediated diseases and autoimmune diseases (Zeng et al., 2017). LPC has been shown to be a chemotactic compound that may amplify inflammation in the epidermis (Ottas et al., 2017). In osteoarthritis, the conversion of phosphatidylcholine to lysophosphatidylcholine is over-activated and may play a role in the disease (Zhai et al., 2018). Two studies found psoriasis patients had elevated plasma levels of this lipid compared to healthy controls, suggesting a possible clinically relevant role in psoriatic disease (Li et al., 2019; Zeng et al., 2017). Sphingomyelin (d18:1/16:0), a sphingolipid and component of animal cell membranes was another confirmed compound discriminating low versus high PsA disease activity (Chakraborty & Jiang, 2013). Increased serum concentrations of sphingolipids have been detected in psoriasis patients with severe disease activity (Burger et al*.*, 2023).

There are many aspects of the untargeted metabolomics workflow that posed a challenge in our research study. We selected inclusion and exclusion criteria for patient samples to maximize our sample size, while limiting sample heterogeneity. The serum samples in our study were selected from PsA patients not previously treated with biologics and with no active infection or history of cancer. In order to achieve this large sample-size research endeavor, the samples were collected from patients at appointments in the PsA clinic over the course of decades and stored in our biobank at − 80 °C. Several factors related to the individual and the environment that were not controlled for, may have contributed to the heterogeneity within the sample population. In our study, the three groups were not age and sex matched, because of the assumption that our large sample size would account for any slight differences between the groups. There were also slight differences in the percentage of patients that were receiving other treatment regimens such as NSAIDs, steroids and DMARDs, between the three groups. We chose to not exclude patients with comorbidities because we wanted the data to be generalizable to the majority of PsA patients, many of whom have said comorbidities. For example, 4% of patients in the high disease activity group had vascular disease, compared to no patients in the low and moderate disease activity groups. We do acknowledge however this can be considered a limitation of our study. We also did not adjust the machine learning methods for comorbidities or treatment regimens, at this discovery stage. A validation study is needed to confirm the findings we have presented.

The wide variety of tools required to analyze data and the assortment of software platforms available to perform these steps, make it challenging to harmonize instrumental acquisition and data analysis in metabolomics studies. In this study, we employed LC-HRMS due to its high sensitivity and comprehensive metabolite coverage, compared to nuclear magnetic resonance approaches (Emwas, 2015; Koussiouris et al., 2021). LC-HRMS analysis was run in positive mode followed by negative mode. Although polarity switching would have achieved shorter instrument acquisition time, we opted to not use this method because it can cause loss of information especially for compounds that can only be analyzed in one mode. To prepare our samples for LC-HRMS analysis, we used a non-exhaustive SPME method that was configured with a thin film device coated with PS-DVB and HLB particles to allow for extraction of a wide range of analytes with a more streamlined, automated workflow (Liu et al., 2020; Vuckovic, 2012, 2013).

Furthermore, we employed pooled quality control samples to correct for time-dependent batch effects and account for signal differences across the long instrumental run. All data was pre-processed on Compound Discoverer and analyzed using predictive feature analysis with multiple classifiers. Depending on the classifier used and the number of features included in the model, the ability of the model to predict disease activity changed. This is to be expected because of the features used and inherent differences of the classifiers. To ensure that our candidate biomarkers were robust across many classifiers, we reported features that overlapped across the models that passed our arbitrary AUC threshold of 0.7.

In addition, identification of features remains one of the key limitations of untargeted metabolomics studies. In order to overcome this bottleneck, we employed four databases to annotate features based on m/z and three databases to annotate compounds from MS/MS spectra. We were successful in tentatively identifying 45% features included in our models, and of those, we were able to confirm 23% of compounds using MS/MS spectra. Further targeted analysis may help achieve greater metabolite identification. We focused on measuring disease activity in this study based on the PASDAS, which is a composite disease activity score considering clinical assessment by a rheumatologist, patient reported outcomes and routine laboratory methods. Future studies should strive to investigate metabolites associated with subgroups of patients with PsA including ultrasonography-defined joint inflammation patterns, skin disease activity, treatment response and comorbidities.

## Conclusion

This study employed an SPME-LC-HRMS method to identify metabolites associated with PsA disease activity. Based on the data presented herein, several lipids and other metabolites when combined in various classifying models could predict high disease activity from both low and moderate disease activity with clinically sufficient sensitivity and specificity. Lipids of key interest included lysophosphatidylcholine and sphingomyelin. Many of these compounds have been suggested to be of interest in previous metabolomics studies investigating PsA, psoriasis and RA. Quantitative MS assays based on selected reaction monitoring, are required to quantify the candidate biomarkers identified. The use of stable isotope-labeled internal standards would allow for absolute quantification and potential clinical translation of these metabolites as diagnostic assays.

### Supplementary Information

Below is the link to the electronic supplementary material.

Supplementary file1 (DOCX 30 kb)

Supplementary file2 (PPTX 1981 kb)

## Data Availability

The metabolomics and metadata reported in this paper are available via MassIVE (https://massive.ucsd.edu) study identifier MSV000092699. The databases reported in this study are accessible via the references in the manuscript.

## Declarations

### Research involving in human and animal participants

All procedures performed in studies involving human participants were in accordance with the ethical standards of the institutional and/or national research committee and with the 1964 Helsinki declaration and its later amendments or comparable ethical standards. Informed consent was obtained from all individual participants included in the study.
