# Supplementary material for: Classifying patients with psoriatic arthritis according to their disease activity status using serum metabolites and machine learning
Source: Metabolomics. 2024 Jan 24;20(1):17. doi: 10.1007/s11306-023-02079-7 (PMC10810020; doi:10.1007/s11306-023-02079-7)
Supplement: Supplementary file 1 — Supplementary file1 (DOCX 30 kb) [file 11306_2023_2079_MOESM1_ESM.docx]

**Online Resource 1**

**Classifying Patients with Psoriatic Arthritis According to their Disease Activity Status using Serum Metabolites and Machine Learning**

John Koussiouris^1,2^, Nikita Looby^1^, Max Kotlyar^3,4^, Vathany Kulasingam^2,5^, Igor Jurisica^3,4,6,7^ and Vinod Chandran^1,2,8,9*^

^1^ Schroeder Arthritis Institute, Krembil Research Institute, University Health Network, Toronto, Canada

^2^ Department of Laboratory Medicine and Pathobiology, University of Toronto, Toronto, Canada
^3^ Osteoarthritis Research Program, Division of Orthopedic Surgery, Schroeder Arthritis Institute, University Health Network, Toronto, Canada
^4^ Data Science Discovery Centre for Chronic Diseases, Krembil Research Institute, University Health Network, Toronto, Canada

^5^ Division of Clinical Biochemistry, Laboratory Medicine Program, University Health Network, Toronto, Canada

^6^ Department of Computer Science, University of Toronto, Toronto, Canada
^7^ Department of Medical Biophysics, University of Toronto, Toronto, Canada
^8^ Division of Rheumatology, Department of Medicine, University of Toronto, Toronto, Canada
^9^ Institute of Medical Science, University of Toronto, Toronto, Canada

*Corresponding author: vinod.chandran@uhn.ca

Table 1: Liquid chromatographic gradient used for separation in positive mode.

| Time (min) | % Mobile phase B  (methanol + 0.1 % formic acid) |
| --- | --- |
| 0 | 5 |
| 1 | 5 |
| 20 | 100 (curve 3) |
| 22.5 | 100 |
| 25 | 5 |
| 30 | 5 |

Table 2: Liquid chromatographic gradient used for separation in negative mode.

| Time (min) | % Mobile phase B  (methanol) |
| --- | --- |
| 0 | 0 |
| 2 | 0 |
| 12 | 100 (curve 3) |
| 15 | 100 |
| 18 | 0 |
| 20 | 0 |

Table 3: Tentatively identified compounds in the top performing predictive models for low vs high disease activity.

| m/z | RT (min) | Tentative ID | Adduct | Biochemical Importance |
| --- | --- | --- | --- | --- |
| 242.1302 | 4.07 | 2-(tert-butylamino)-1-(3-chlorophenyl)propan-1-ol | [M+H]^+^ | Phenylpropane. It has been detected in a few different foods. |
| 291.1200 | 8.54 | 2-(2-Carboxyethyl)-4-methyl-5-pentyl-3-furoic acid | [M+Na]^+^ | Furanoid fatty acid. |
| 313.1543 | 3.77 | Phenylalanylphenylalanine | [M+H]^+^ | Peptide made of two phenylalanine molecules. |
| 369.3513 | 19.24 | (5Z,7E)-9,10-Secocholesta-5,7,10-triene | [M+H] ^+^ | Secosteroid. |
| 429.3723 | 19.32 | (-)-Cholesteryl acetate | [M+H]^+^ | Normal human cholesteryl ester. |
| 758.5687 | 19.89 | 1-Palmitoyl-2-linoleoyl-glycero-3-phosphocholine | [M+H]^+^ | N/A |
| 235.0980 | 5.53 | Ethylvanillin isobutyrate | [M-H]^-^ | Phenol ester. |
| 239.0929 | 6.25 | 2-(Hydroxyamino)-1-methyl-6-phenylimidazo[4,5-b]pyridine | [M-H]^-^ | Phenylpyridine. Considered part of the human exposome. |
| 311.1408 | 4.37 | Phenylalanylphenylalanine | [M-H]^-^ | Peptide made of two phenylalanine molecules. |
| 539.2506 | 6.00 | Tetrahydroaldosterone-3-glucuronide | [M-H]^-^ | Natural human metabolite of Tetrahydroaldosterone. |
| 541.2662 | 5.93 | Cortolone-3-glucuronide | [M-H]^-^ | Natural human metabolite of cortolone. |
| 802.5618 | 13.19 | PE(PGF1alpha/P-18:0) | [M-H]^-^ | Oxidized phosphatidylethanolamine. |
| 826.5619 | 13.16 | PC(20:4(8Z,11Z,14Z,17Z)-2OH(5S,6R)/17:0) | [M-H]^-^ | Oxidized phosphatidylcholine. |
| 830.5933 | 13.6 | PE(18:1(12Z)-2OH(9,10)/22:1(13Z)) | [M-H]^-^ | Oxidized phosphatidylethanolamine. |
| 870.5493 | 13.19 | PC(PGF1alpha/DiMe(9,3)) | [M-H]^-^ | Oxidized phosphatidylcholine. |

Table 4: Tentatively identified compounds in the top performing predictive models for moderate vs high disease activity.

| m/z | RT (min) | Tentative ID | Adduct | Biochemical Importance |
| --- | --- | --- | --- | --- |
| 181.0859 | 6.81 | 4-Ethoxy-m-anisaldehyde | [M+H]^+^ | Benzoyl derivative. |
| 263.0887 | 6.81 | Ethyl 3,4,5-trimethoxybenzoate | [M+H]^+^ | Gallic acid derivative. |
| 242.1302 | 4.07 | 2-(tert-butylamino)-1-(3-chlorophenyl)propan-1-ol | [M+H]^+^ | Phenylpropane. It has been detected in a few different foods. |
| 780.5532 | 19.47 | PC(18:3(9Z,12Z,15Z)/18:2(9Z,12Z)) | [M+H]^+^ | Phosphatidylcholine. |
| 806.5687 | 19.80 | PC(16:0/22:6(4Z,7Z,10Z,13Z,16Z,19Z)) | [M+H]^+^ | Phosphatidylcholine. |
| 808.5844 | 20.00 | PC(16:0/22:5(7Z,10Z,13Z,16Z,19Z)) | [M+H]^+^ | Phosphatidylcholine. |
| 414.2999 | 8.10 | (9Z,12Z,15Z)-N-[2-(3,4-Dihydroxyphenyl)ethyl]-9,12,15-octadecatrienamide | [M+H]^+^ | Fatty acyl. |
| 701.5587 | 18.72 | N-(9Z-hexadecenoyl) sphingosine-1-phosphocholine | [M+H]^+^ | Sphingolipid found in animal cell membranes. |
| 279.1701 | 3.55 | Leucyl-phenylalanine | [M+H]^+^ | Dipeptide. |
| 201.0546 | 7.92 | 5-Methylangelicin | [M+H]^+^ | Furanocoumarin. |
| 286.1436 | 7.93 | Hydromorphone | [M+NH4]^+^ | Opioid analgesic derived from morphine. |
| 500.2769 | 11.71 | LysoPE(20:5(5Z,8Z,11Z,14Z,17Z)/0:0) | [M+H]^+^ | Lysophosphatidylethanolamines are approved for agricultural use. |
| 542.3240 | 11.35 | LysoPC(20:5(5Z,8Z,11Z,14Z,17Z)/0:0) | [M+H]^+^ | Lysophosphatidylcholine. Pro-inflammatory properties and pathological component in atherosclerotic lesions. |
| 729.5900 | 19.56 | SM(d18:1/18:1(11Z)) | [M+H]^+^ | Sphingolipid found in animal cell membranes. |
| 813.6838 | 21.45 | SM(d18:1/24:1(15Z)) | [M+H]^+^ | Sphingolipid found in animal cell membranes. |
| 133.0661 | 5.53 | Cinnamyl alcohol | [M-H]^-^ | Cinnamyl alcohol. |
| 239.0929 | 6.25 | 2-(Hydroxyamino)-1-methyl-6-phenylimidazo[4,5-b]pyridine | [M-H]^-^ | Phenylpyridine |
| 311.1408 | 4.37 | Phenylalanylphenylalanine | [M-H]^-^ | Peptide made of two phenylalanine molecules. |
| 369.1746 | 8.44 | Androsterone sulfate | [M-H]^-^ | 5-alpha-reduced androgen metabolite. |
| 407.2808 | 8.10 | Cholic acid | [M-H]^-^ | Major primary bile acid. Facilitates fat absorption and cholesterol excretion. |
| 448.3075 | 8.12 | Glycochenodeoxycholic acid | [M-H]^-^ | Bile salt. Acts as a detergent to solubilize fats for absorption. |
| 269.0549 | 7.17 | Leflunomide | [M-H]^-^ | DMARD (disease-modifying antirheumatic drug). |
| 381.1561 | 6.64 | Ibuprofen glucuronide | [M-H]^-^ | o-Glucuronide. |
| 410.0434 | 6.24 | Carboxycelecoxib | [M-H]^-^ | Metabolite of celecoxib, a sulfa non-steroidal anti-inflammatory drug (NSAID). |
| 476.2787 | 9.49 | LysoPE(18:2(9Z,12Z)/0:0) | [M-H]^-^ | Lysophosphatidylethanolamine (LPE). LPEs are approved for outdoor and indoor agricultural use. |
| 539.2506 | 6.00 | Tetrahydroaldosterone-3-glucuronide | [M-H]^-^ | N/A |

Table 5: Tentatively identified compounds in the top performing predictive models for low vs moderate and high disease activity.

| m/z | RT (min) | Tentative ID | Adduct | Biochemical Importance |
| --- | --- | --- | --- | --- |
| 159.0917 | 2.33 | 1,5-Naphthalenediamine | [M+H]^+^ | Naphthalene. |
| 211.0822 | 1.84 | 1,3,7-Trimethyluric acid | [M+H]^+^ | Derivative of uric acid. |
| 246.1697 | 2.54 | 2-methylbutyrylcarnitine | [M+H]^+^ | Short chain acylcarnitine. |
| 279.1701 | 3.55 | Leucyl-phenylalanine | [M+H]^+^ | Dipeptide. |
| 288.2168 | 4.78 | Octanoylcarnitine | [M+H]^+^ | Medium chain acylcarnitine. |
| 297.1693 | 7.08 | 3-Hydroxydodeca-5,7-dienoylcarnitine |  | Medium chain acylcarnitine. |
| 429.3723 | 19.32 | (-)-Cholesteryl acetate | [M+H]^+^ | Normal human cholesteryl ester. |
| 732.5533 | 19.65 | PE-NMe2(18:1(9Z)/15:0) |  | Dimethylphosphatidylethanolamine. |
| 758.5687 | 19.89 | 1-Palmitoyl-2-linoleoyl-glycero-3-phosphocholine | [M+H]^+^ | N/A |
| 760.5844 | 20.34 | PC(16:0/18:1(9Z)) | [M+H]^+^ | Phosphatidylcholine. |
| 786.6000 | 20.58 | (2R)-2,3-Bis[(9E)-9-octadecenoyloxy]propyl 2-(trimethylammonio)ethyl phosphate | [M+H]^+^ | N/A |
| 788.6157 | 20.99 | PC(18:1(9Z)/18:0) | [M+H]^+^ | Phosphatidylcholine. |
| 812.6157 | 20.80 | PC(18:0/20:3(8Z,11Z,14Z)) | [M+H]^+^ | Phosphatidylcholine. |
