# Supplementary material for: Classifying patients with psoriatic arthritis according to their disease activity status using serum metabolites and machine learning
Source: Metabolomics. 2024 Jan 24;20(1):17. doi: 10.1007/s11306-023-02079-7 (PMC10810020; doi:10.1007/s11306-023-02079-7)
Supplement: Supplementary file 2 — Supplementary file2 (PPTX 1981 kb) [file 11306_2023_2079_MOESM2_ESM.pptx]

## Slide 1
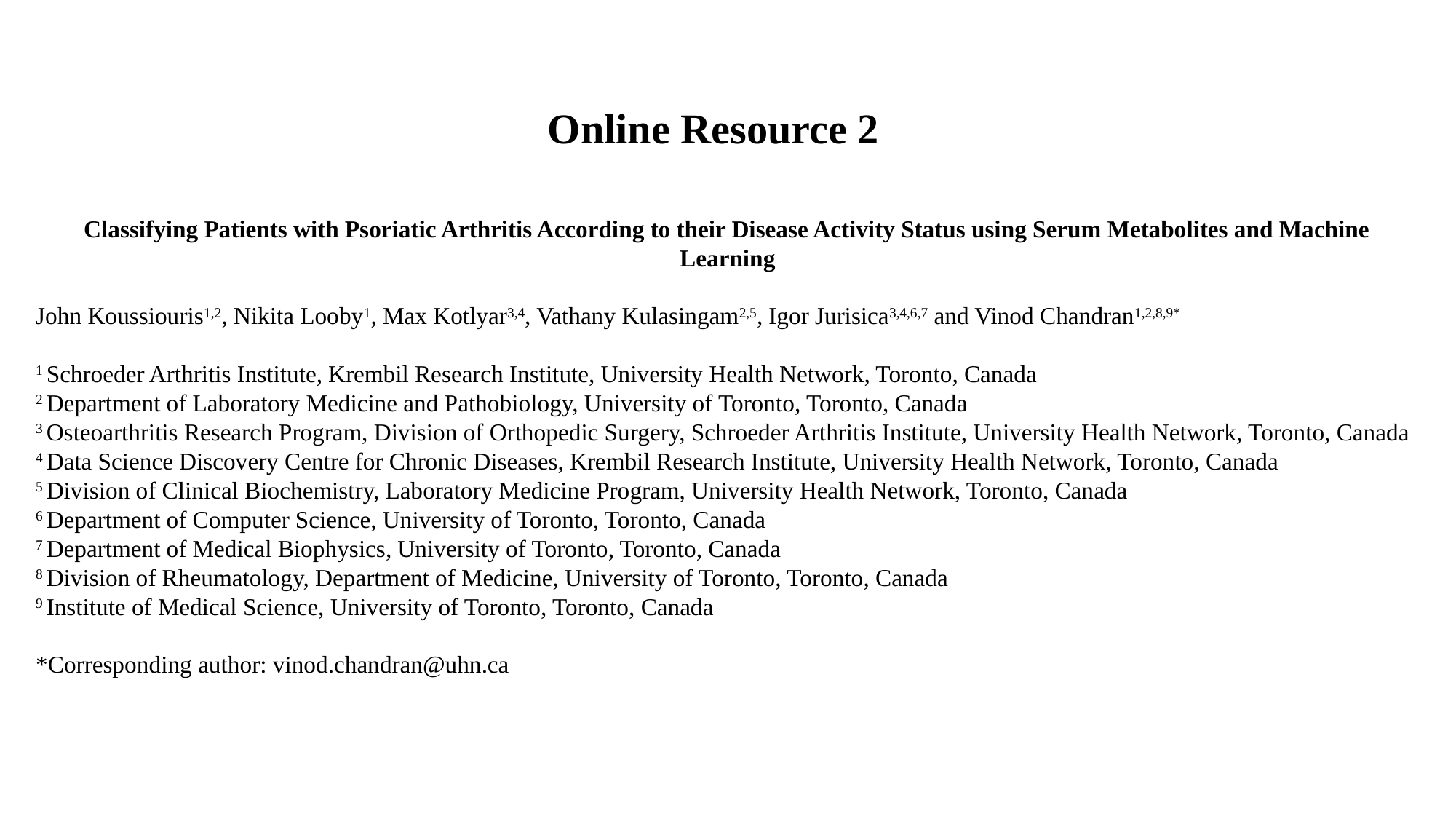

Online Resource 2
Classifying Patients with Psoriatic Arthritis According to their Disease Activity Status using Serum Metabolites and Machine Learning
John Koussiouris1,2, Nikita Looby1, Max Kotlyar3,4, Vathany Kulasingam2,5, Igor Jurisica3,4,6,7 and Vinod Chandran1,2,8,9*
1 Schroeder Arthritis Institute, Krembil Research Institute, University Health Network, Toronto, Canada
2 Department of Laboratory Medicine and Pathobiology, University of Toronto, Toronto, Canada
3 Osteoarthritis Research Program, Division of Orthopedic Surgery, Schroeder Arthritis Institute, University Health Network, Toronto, Canada
4 Data Science Discovery Centre for Chronic Diseases, Krembil Research Institute, University Health Network, Toronto, Canada
5 Division of Clinical Biochemistry, Laboratory Medicine Program, University Health Network, Toronto, Canada
6 Department of Computer Science, University of Toronto, Toronto, Canada
7 Department of Medical Biophysics, University of Toronto, Toronto, Canada
8 Division of Rheumatology, Department of Medicine, University of Toronto, Toronto, Canada
9 Institute of Medical Science, University of Toronto, Toronto, Canada
*Corresponding author: vinod.chandran@uhn.ca

## Slide 2
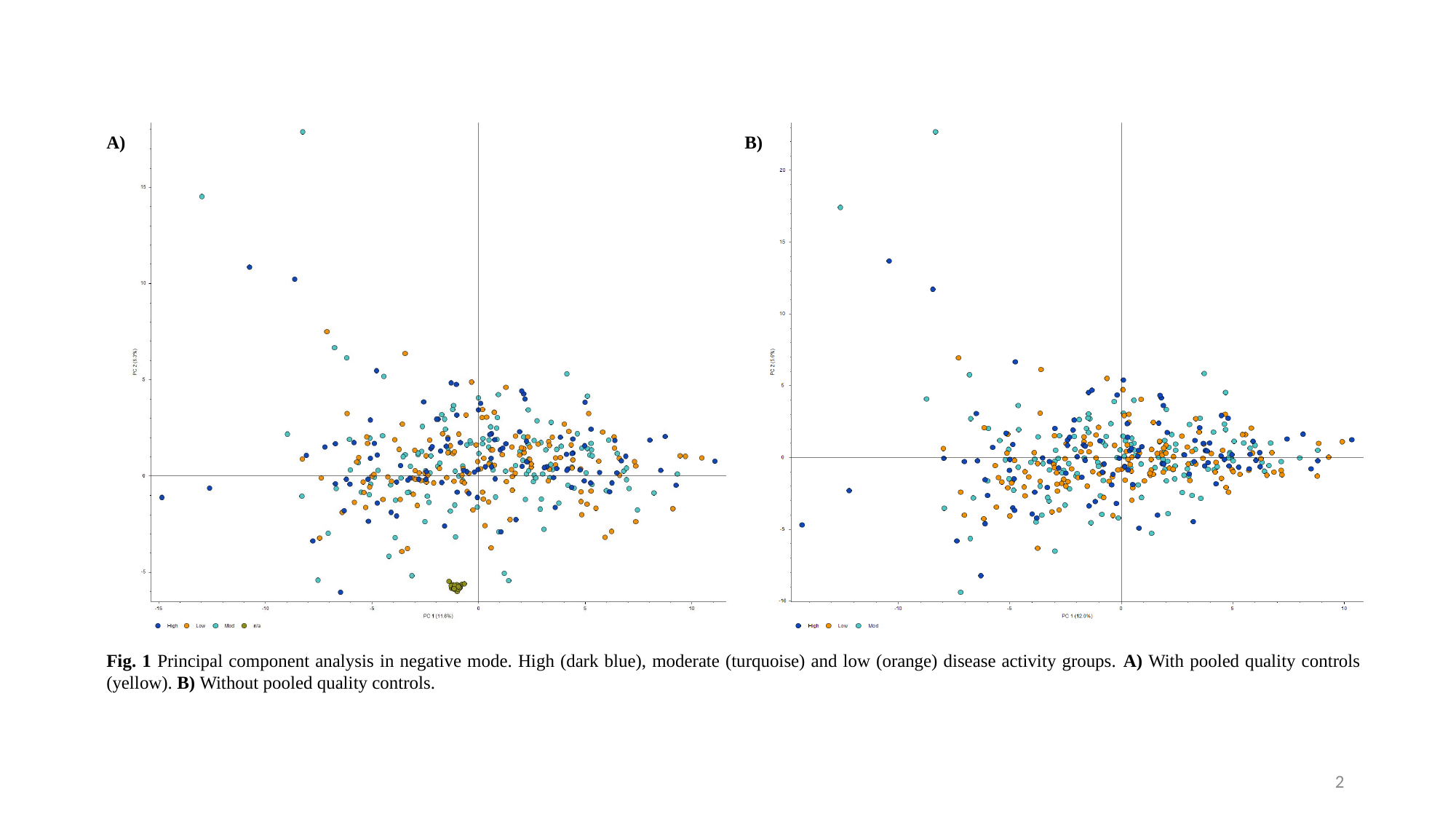

B)
A)
Fig. 1 Principal component analysis in negative mode. High (dark blue), moderate (turquoise) and low (orange) disease activity groups. A) With pooled quality controls (yellow). B) Without pooled quality controls.
2

## Slide 3
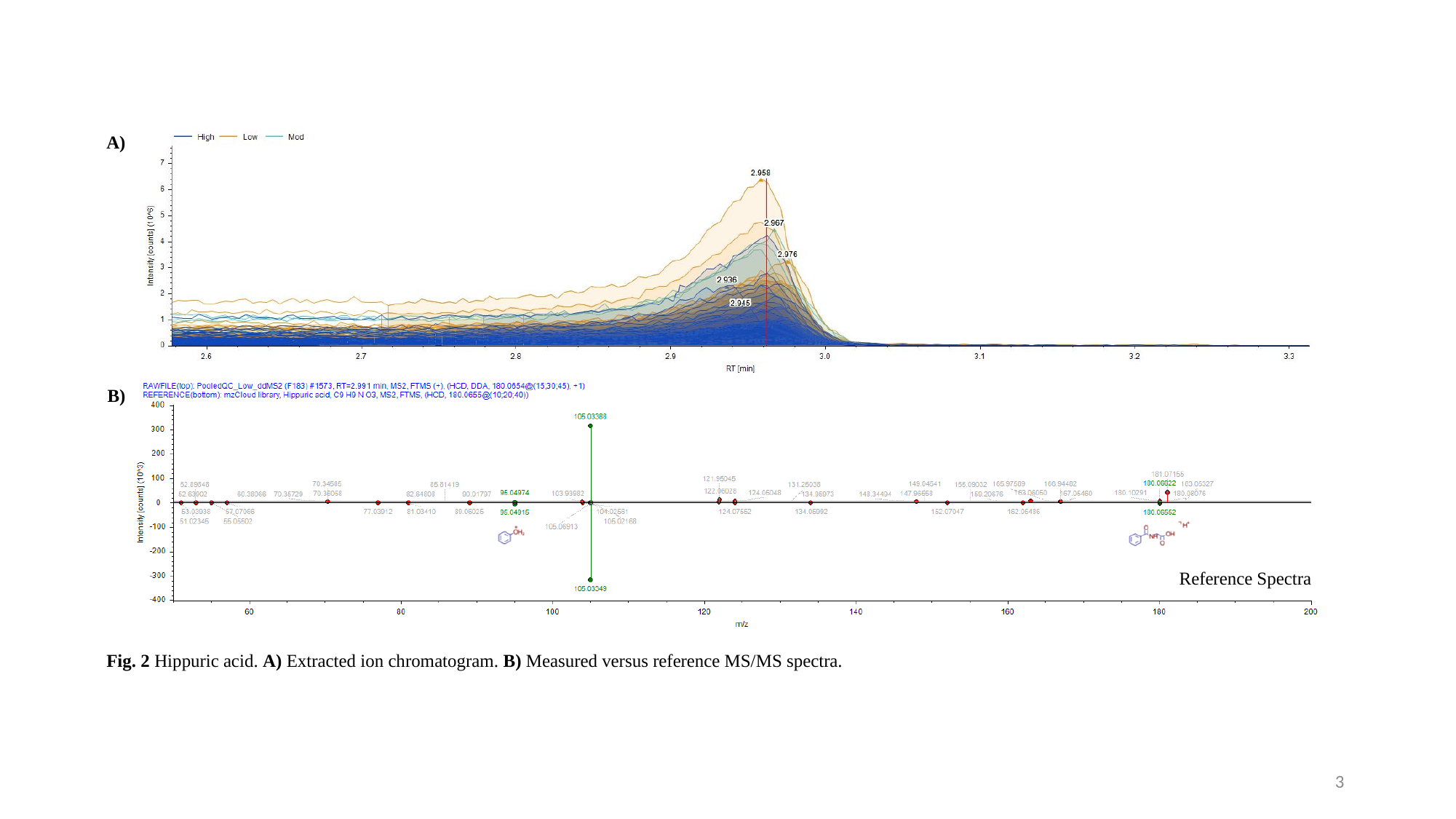

A)
B)
Reference Spectra
Fig. 2 Hippuric acid. A) Extracted ion chromatogram. B) Measured versus reference MS/MS spectra.
3

## Slide 4
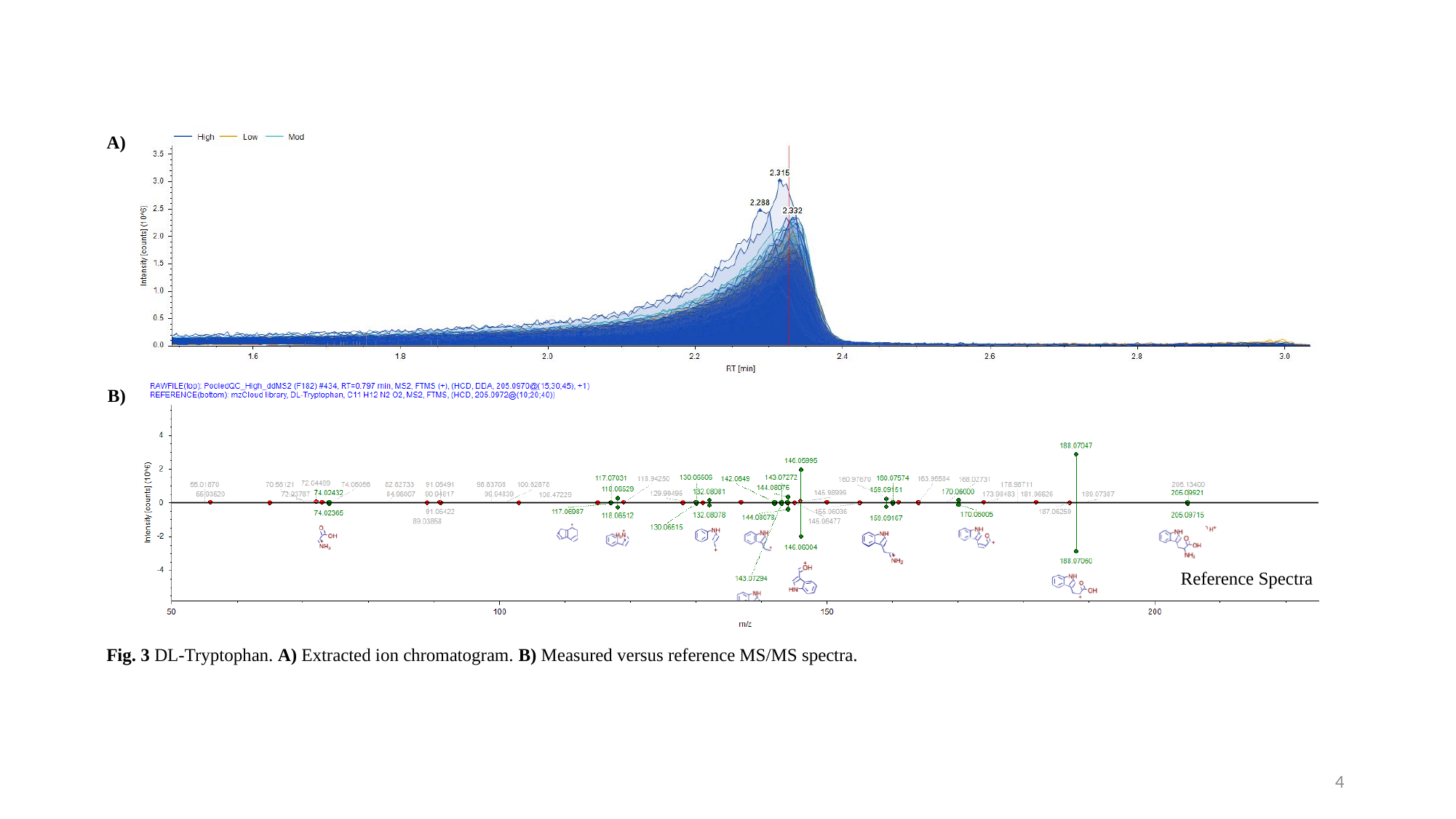

A)
B)
Reference Spectra
Fig. 3 DL-Tryptophan. A) Extracted ion chromatogram. B) Measured versus reference MS/MS spectra.
4

## Slide 5
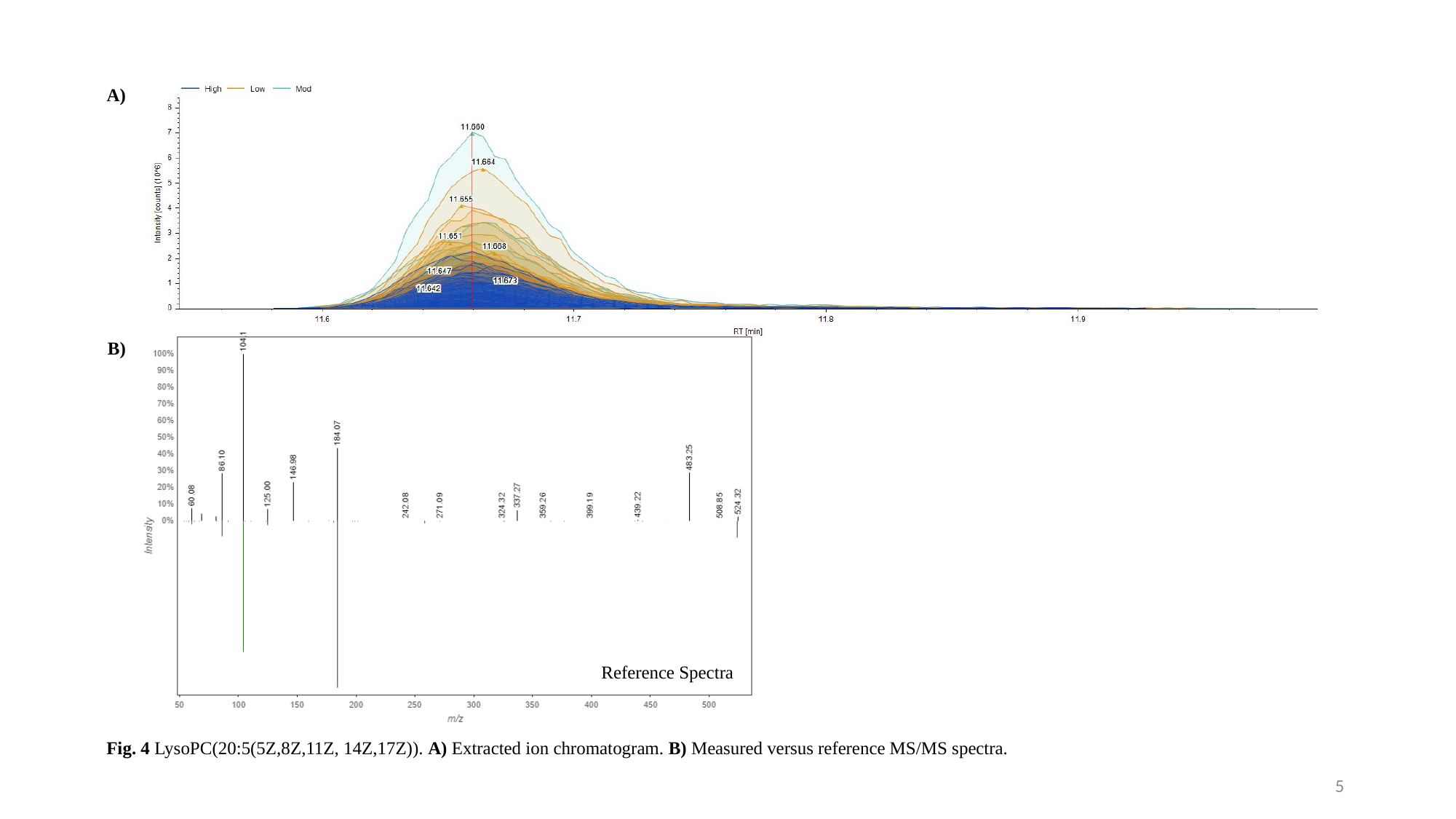

A)
B)
Reference Spectra
Fig. 4 LysoPC(20:5(5Z,8Z,11Z, 14Z,17Z)). A) Extracted ion chromatogram. B) Measured versus reference MS/MS spectra.
5

## Slide 6
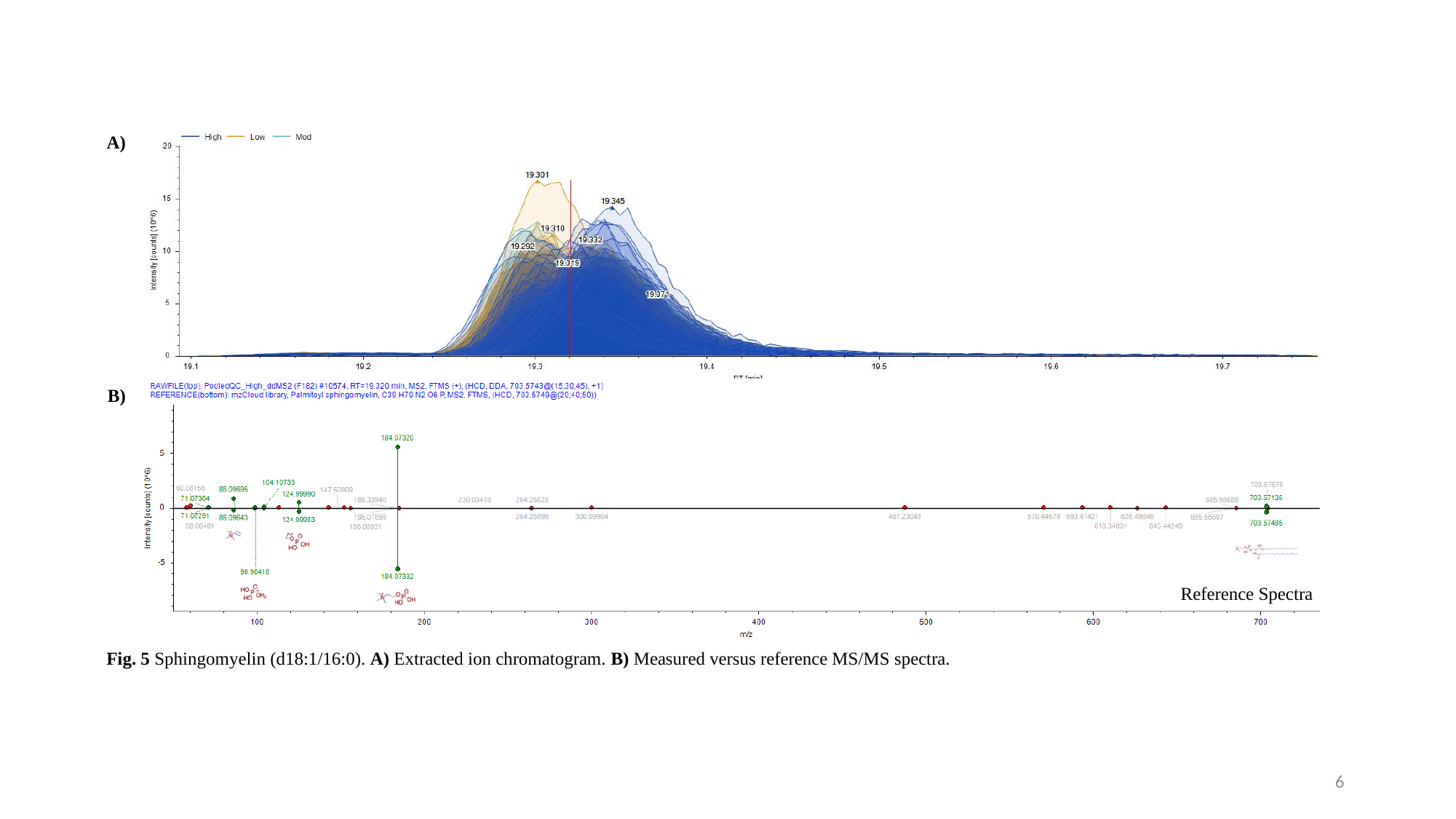

A)
B)
Reference Spectra
Fig. 5 Sphingomyelin (d18:1/16:0). A) Extracted ion chromatogram. B) Measured versus reference MS/MS spectra.
6

## Slide 7
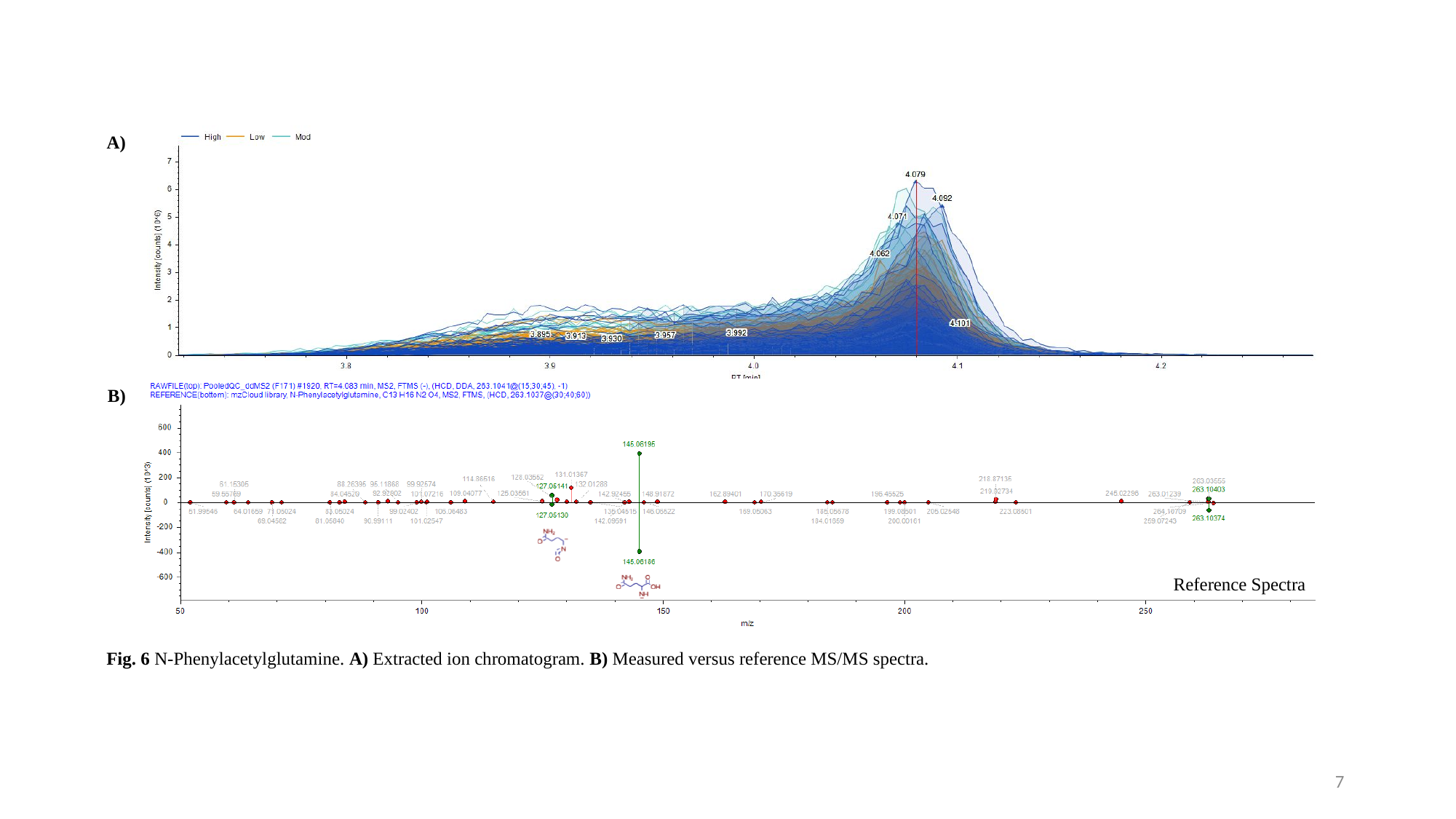

A)
B)
Reference Spectra
Fig. 6 N-Phenylacetylglutamine. A) Extracted ion chromatogram. B) Measured versus reference MS/MS spectra.
7

## Slide 8
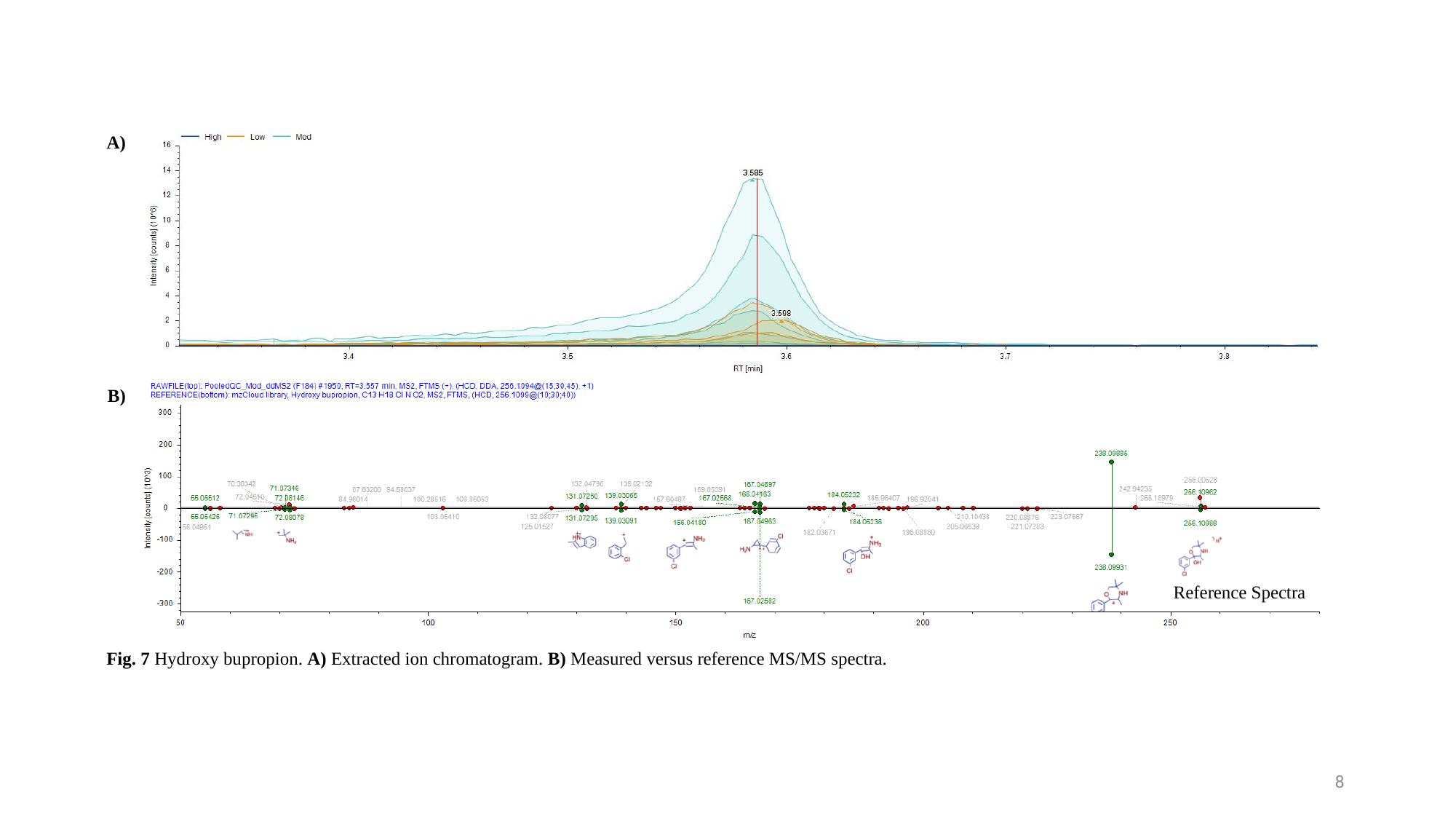

A)
B)
Reference Spectra
Fig. 7 Hydroxy bupropion. A) Extracted ion chromatogram. B) Measured versus reference MS/MS spectra.
8

## Slide 9
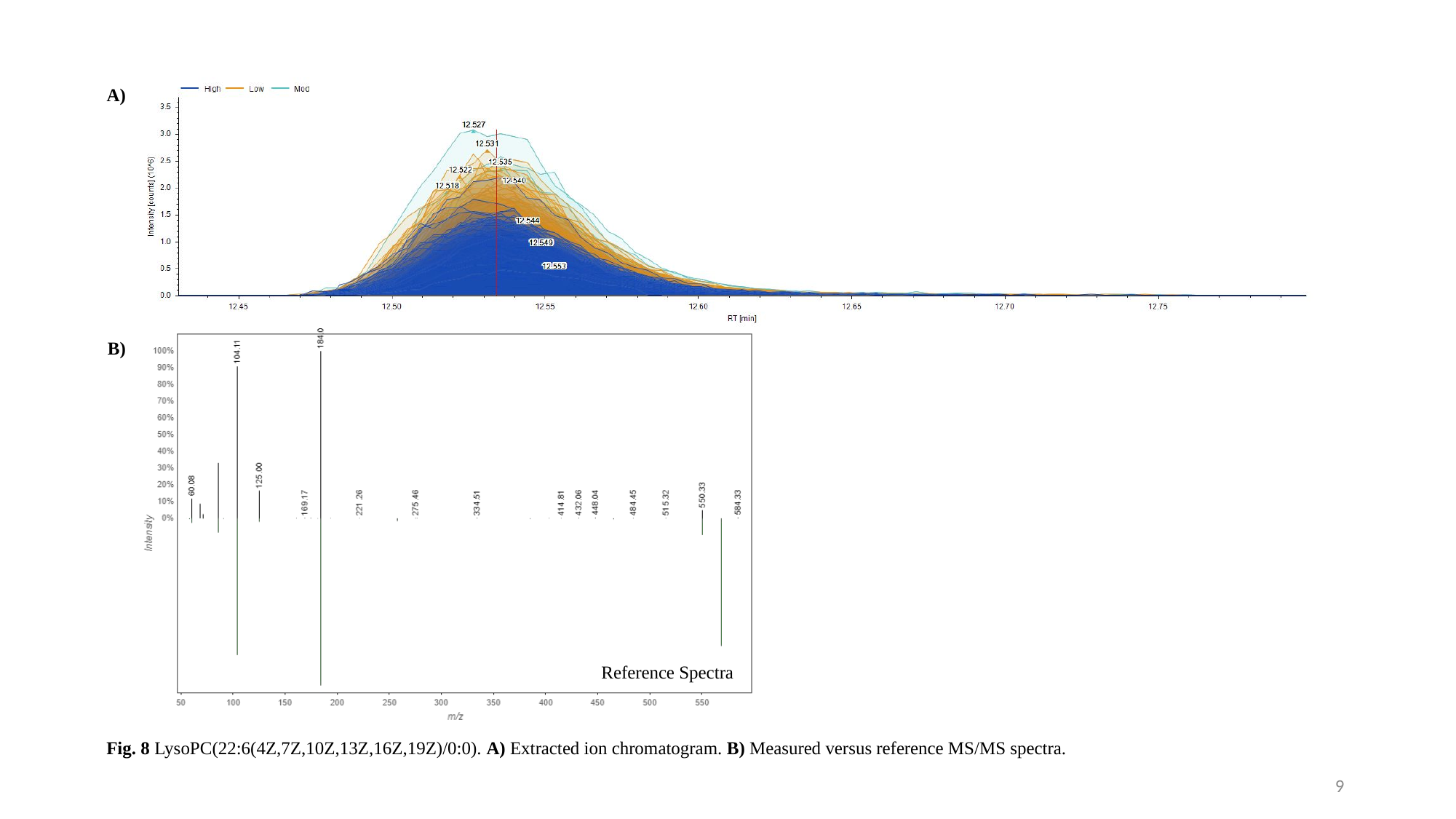

A)
B)
Reference Spectra
Fig. 8 LysoPC(22:6(4Z,7Z,10Z,13Z,16Z,19Z)/0:0). A) Extracted ion chromatogram. B) Measured versus reference MS/MS spectra.
9

## Slide 10
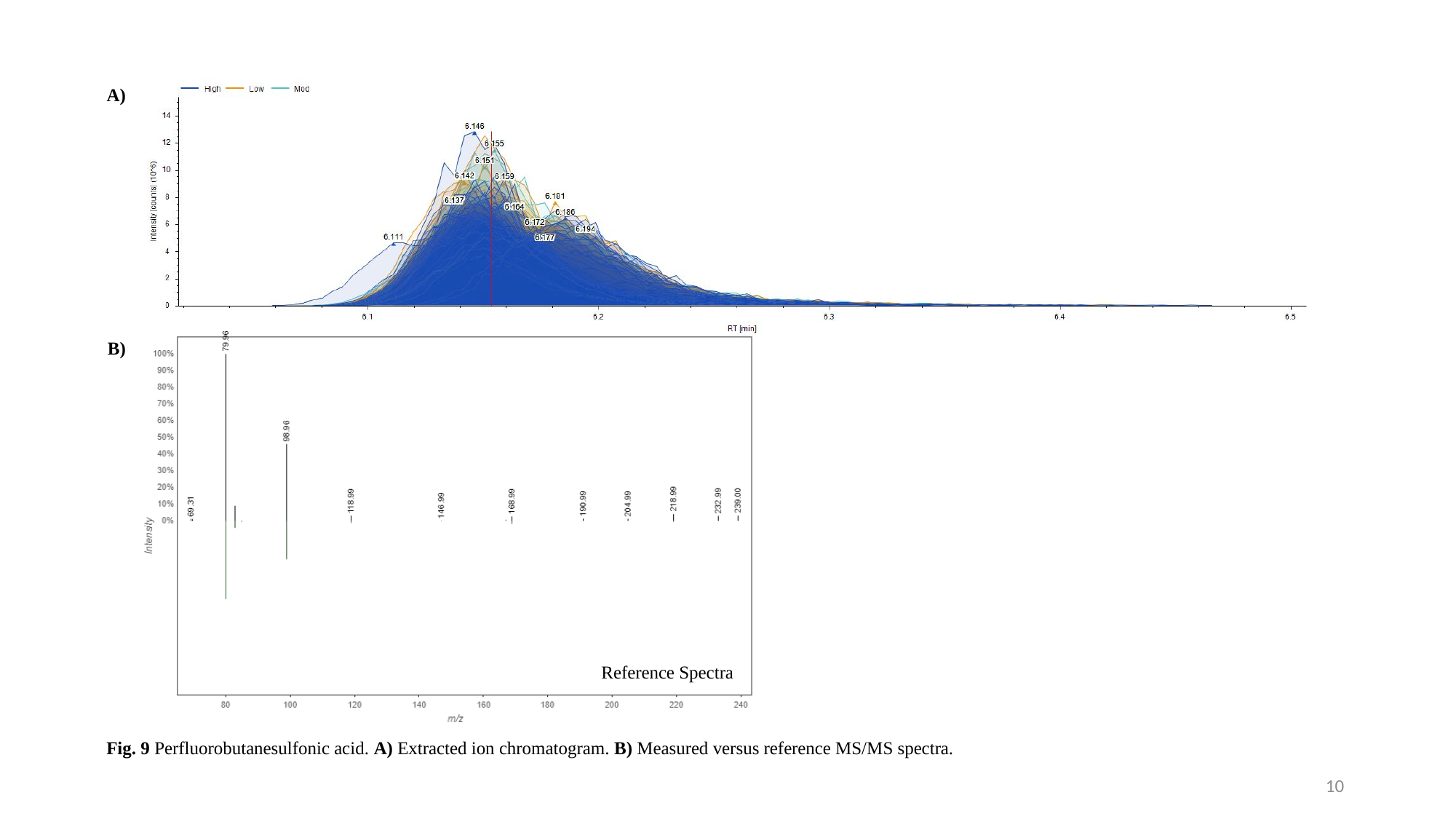

A)
B)
Reference Spectra
Fig. 9 Perfluorobutanesulfonic acid. A) Extracted ion chromatogram. B) Measured versus reference MS/MS spectra.
10
